# Assessment of albumin-related inflammatory indices in preeclampsia and their relationship with composite adverse perinatal outcomes

**DOI:** 10.1186/s12884-026-09208-9

**Published:** 2026-05-04

**Authors:** Aykut Kından, Goncagül Kından, Dilara Sarikaya Kurt, Ayberk Çakır, Özgür Volkan Akbulut, Şevki Çelen

**Affiliations:** 1 Department of Obstetrics and Gynecology, Nurdağı State Hospital, Gaziantep, Turkey; 2 Department of Obstetrics and Gynecology, Atatürk Sanatoryum Training and Research Hospital, Ankara, Turkey; 3 Department of Obstetrics and Gynecology, Mudanya State Hospital, Bursa, Turkey; 4 Department of Obstetrics and Gynecology, Acıbadem University Atakent Hospital, Istanbul, Turkey; 5 Department of Perinatology, Etlik City Hospital, Ankara, Turkey

**Keywords:** Preeclampsia, Composite adverse perinatal outcome, Blood urea nitrogen-to-albumin ratio (BAR), Albumin-based inflammatory indices, Neonatal outcomes

## Abstract

**Background:**

Preeclampsia is characterized by systemic inflammation, endothelial dysfunction, and multi-organ involvement, contributing to increased risk of adverse perinatal outcomes. Albumin-based inflammatory and nutritional indices have recently gained interest as easily accessible biomarkers in high-risk pregnancies. This study aimed to compare albumin-related indices between preeclamptic and normotensive pregnancies and to evaluate their predictive value for composite adverse perinatal outcomes (CAPO) in preeclampsia.

**Methods:**

This retrospective case–control study included 186 preeclamptic patients and 186 healthy controls matched for maternal age, BMI, gravidity, and gestational age. Laboratory parameters measured at delivery and calculated indices (NAR, FAR, sACR, HALP, and BAR) were compared. The preeclampsia cohort was further classified into CAPO (+) and CAPO (–) groups. Logistic regression models were used to identify independent predictors of CAPO, and receiver operating characteristic (ROC) analysis assessed predictive performance.

**Results:**

Albumin-based indices differed significantly between preeclamptic and healthy pregnancies, with higher NAR, FAR, and BAR values and lower sACR levels in the preeclampsia group (all *p* < 0.001). Among preeclamptic patients, CAPO developed in 44.1% (*n* = 82). CAPO (+) patients demonstrated higher NAR, BAR, and HALP values and lower sACR levels compared to CAPO (–) patients. In multivariable logistic regression, only the blood urea nitrogen-to-albumin ratio (BAR) remained independently associated with CAPO (aOR = 1.157, *p* = 0.015). BAR demonstrated moderate discriminative performance for CAPO (AUC = 0.718; cut-off > 0.61; sensitivity 67.1%; specificity 69.2%; *p* < 0.001).

**Conclusion:**

All albumin-based indices showed significant alterations in preeclampsia; however, only BAR independently predicted adverse perinatal outcomes. BAR is a simple, inexpensive biomarker derived from routine laboratory parameters and may support risk stratification and neonatal outcome prediction in preeclamptic pregnancies. Prospective multicenter studies are warranted to validate its clinical applicability.

## Background

Preeclampsia is a serious obstetric disorder characterized by the onset of hypertension after 20 weeks of gestation, accompanied by proteinuria or evidence of multi-organ involvement [1]. It affects approximately 2–8% of pregnancies worldwide [2, 3]. Reported risk factors include nulliparity, obesity, advanced maternal age, chronic hypertension, and genetic predisposition [3–5]. The condition can lead to substantial morbidity for both the mother and the fetus [6–8]. Maternal complications include postpartum hemorrhage, placental abruption, and prolonged hospitalization [6]; whereas neonatal complications often involve preterm birth, low birth weight, the need for intensive neonatal care, and increased perinatal mortality [7, 8].

The occurrence of adverse neonatal outcomes in preeclampsia is associated with increased maternal inflammation, uteroplacental insufficiency, and subsequent fetal hypoxia [9–12]. Endothelial dysfunction, increased oxidative stress, and an inflammatory response disrupt placental perfusion, increasing the risk of intrauterine growth restriction, preterm birth, and neonatal morbidity [9–11]. Some biomarkers (e.g., sFlt-1, PlGF) have been investigated in the literature for predicting adverse neonatal outcomes [7]; however, they have not become widespread in clinical use due to high cost and limited availability.

Therefore, in recent years, composite indices derived from routine laboratory parameters, especially albumin-based ones, have gained attention. Recent studies in obstetric populations have also highlighted the potential clinical utility of inflammation-based laboratory markers in complications such as emergent cerclage, placenta accreta spectrum, and placental abruption [13–15]. Preeclampsia causes endothelial damage [16], leading to decreased serum albumin levels due to liver dysfunction, inflammatory response, and increased vascular permeability. Conversely, neutrophil levels increase due to the inflammatory response, fibrinogen levels increase due to coagulation activity, and urea and creatinine levels increase due to renal dysfunction and catabolism [17, 18]. These biochemical changes, when combined with a decrease in albumin, enhance the clinical significance and discriminatory power of the indices. In this context, NAR reflects neutrophil-dominant systemic inflammation, FAR indicates inflammation-related coagulative activity, BAR and sACR reflect renal/endothelial involvement, and HALP summarizes immuno-nutritional status, all of which are easily calculable indices derived from routine laboratory parameters.

It has been reported that NAR, NPAR, and FAR are associated with disease severity in the context of preeclampsia and may be independent indicators for severe preeclampsia [19, 20]. In studies on placental abruption in the field of obstetrics, albumin-based ratios such as NPAR, NAR, BAR, and sACR have been examined, and it has been reported that these indices offer statistically significant predictive value in predicting placental abruption [15]. On the other hand, the HALP score was found to be decreased in late-onset FGR in the first trimester in relation to obstetric outcomes [21]; it also provided significant confirmatory metrics for predicting preterm birth in the first trimester [22].

In the present study, we aimed to evaluate albumin-related inflammatory indices not only in terms of their differences between preeclamptic and normotensive pregnancies, but also in terms of their potential association with composite adverse perinatal outcomes (CAPO) in preeclampsia. NAR, FAR, sACR, BAR, and HALP were examined within a biologically informed analytical framework. NAR was considered to reflect neutrophil-dominant systemic inflammation, FAR the inflammation–coagulation axis, sACR and BAR renal/endothelial involvement, and HALP the immuno-nutritional status. Because adverse neonatal outcomes in preeclampsia are closely linked to endothelial dysfunction, impaired uteroplacental perfusion, and systemic inflammation, indices incorporating renal and endothelial involvement, particularly BAR and sACR, were considered biologically plausible candidate markers for CAPO. However, as the relative predictive performance of these markers for CAPO has not been clearly established in the literature, the present analysis also included an exploratory component. Accordingly, all indices were evaluated in parallel within a predefined, biologically grounded framework.

## Methods

This retrospective case-control study was conducted at Ankara Etlik City Hospital. The study encompassed preeclamptic patients who were monitored and delivered at our hospital from November 2022 to January 2025, along with healthy controls. Our research has received approval from the institutional ethics committee (Approval number: AEŞH-BADEK2-2025-459) and was carried out in accordance with the Declaration of Helsinki. Because the study is retrospective in nature, informed consent was not obtained.

### Participants

**Fig. 1:**
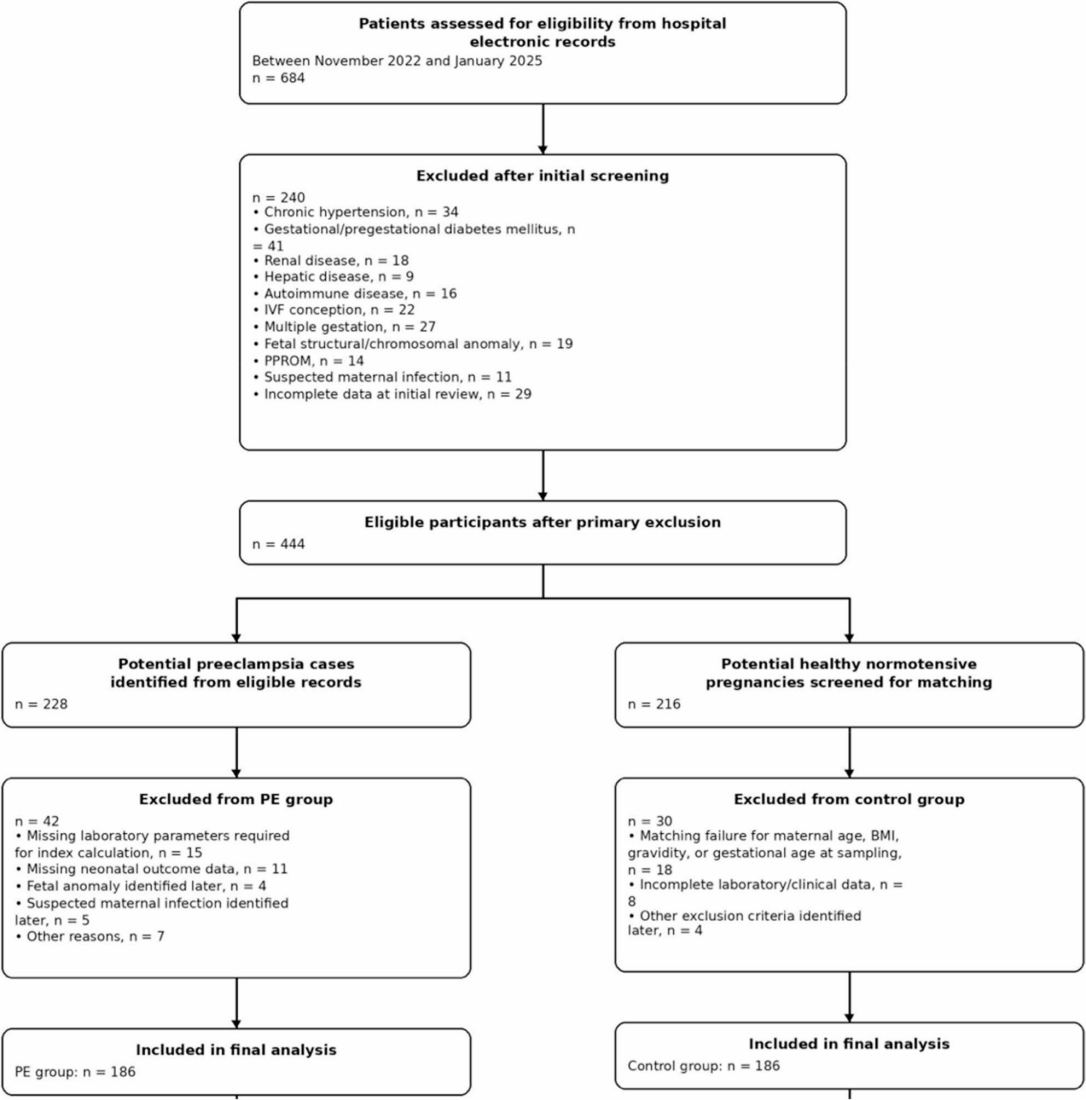
Flow diagram of participant selection for the retrospective case–control study

The study group included patients diagnosed with preeclampsia who were delivered in our hospital. The diagnosis of preeclampsia and severe preeclampsia was made according to the recommendations of the American College of Obstetricians and Gynecologists (ACOG) [1]. The control group consisted of healthy pregnant women in a 1:1 ratio, matched by maternal age, body mass index (BMI), gravidity number, and gestational age at sampling, who were delivered and received postnatal care in our hospital. Participants with known chronic diseases (such as gestational or chronic diabetes mellitus, chronic hypertension, renal disease, hepatic disease, autoimmune disorders, etc.), those who conceived via IVF, multiple gestations, fetal chromosomal or structural abnormalities, preterm prelabor rupture of membranes, and suspected maternal infections were not included in the study. Furthermore, cases with incomplete data were not included in the study. The participant selection process, including initial screening, exclusions with reasons, and final inclusion in the preeclampsia and control groups, is summarized in Fig. 1.

The preeclampsia cohort was categorized into two subgroups: individuals with and without composite adverse perinatal outcomes (CAPO). CAPO was recorded as present if ≥ 1 component occurred: Apgar score at 5 min ≤ 7, fetal or neonatal mortality, neonatal intensive care unit (NICU) admission exceeding 2 days, respiratory distress syndrome (RDS), inotrope requirement, developing sepsis, necessity for mechanical ventilation, and neonatal acidosis.

### Data collection

The hospital’s electronic records were used to access the data of the study participants. These data included maternal demographic and clinical characteristics such as maternal age, BMI, gravida, parity, and gestational week. Among the obstetric variables were the presence of placental abruption, the need for blood product transfusion, and admission to the intensive care unit (ICU). Perinatal variables recorded included gestational age at birth, birth weight, 1st and 5th minute Apgar scores, NICU admission, NICU length of stay (in days), and the presence of RDS, sepsis, the need for mechanical ventilation, inotropic support, and neonatal acidosis.

In this retrospective study, laboratory values ​​in the preeclampsia group were obtained from blood results obtained at the time of delivery. In the control group, healthy pregnant women with blood tests available at the gestational age matched to the preeclamptic women were selected. Complete blood count parameters (hemoglobin, white blood cell count, neutrophil, lymphocyte count, platelet count) and biochemical and coagulation parameters (serum albumin, fibrinogen, creatinine, liver enzymes (AST, ALT), and blood urea nitrogen (BUN) were evaluated. Additionally, composite indices reflecting inflammation and nutritional status were calculated: the neutrophil/albumin ratio (NAR), serum albumin/creatinine ratio (sACR), fibrinogen/albumin ratio (FAR), BUN/albumin ratio (BAR), and HALP (hemoglobin–albumin–lymphocyte–platelet) score.

### Study outcomes

The primary aim of this study is to compare albumin-related indices in preeclamptic patients and healthy pregnant women, and to evaluate whether these indices differ between preeclamptic cases who develop CAPO and those who do not. Additionally, the predictive value of these indices in forecasting CAPO has also been analyzed.

### Sample size calculation

The sample size was calculated using the G*Power 3.1.9.7 software for a comparison of two independent means (t-test). As no previous study with a similar design was available, a moderate effect size (Cohen’s d = 0.5) was assumed. With a significance level of 0.05 and a power of 0.95, a minimum of 105 participants per group (total = 210) was estimated to be sufficient.

### Statistical analysis

Statistical analyses were performed using the IBM SPSS Statistics 27.0 package program. The Kolmogorov-Smirnov and Shapiro-Wilk tests were used to determine whether the continuous variables followed a normal distribution. For the comparison of normally distributed parameters between two independent groups, the independent samples t-test was used, and the results were expressed as mean ± standard deviation (SD). For variables with non-normal distributions, the Mann–Whitney U test was used, and results are presented as median (25th–75th percentile). The relationships between qualitative variables were analyzed using Pearson’s χ² test. Receiver operating characteristic (ROC) curve analysis was performed to assess the predictive power of NAR, sACR, BAR, and HALP scores for CAPO in the preeclampsia cohort. Logistic regression analyses were performed to identify independent predictors of CAPO in preeclampsia. Because NAR, sACR, BAR, and HALP share albumin as a common component, separate multivariable logistic regression models were constructed for each index to reduce potential collinearity and improve interpretability. Because NAR and BAR had relatively small raw values, these variables were multiplied by 10 before logistic regression analysis to improve the interpretability of the odds ratios and confidence intervals. This rescaling did not affect statistical significance, but only changed the unit of interpretation of the reported odds ratios. Statistical significance was defined as a p-value < 0.05.

## Results

A total of 372 pregnant women were included in the study, with 186 in the preeclampsia (PE) group and 186 in the healthy group. A comparison of maternal demographics, clinical outcomes, and neonatal outcomes between the groups is presented in Table 1. No significant differences were found between the two groups in terms of maternal age (*p* = 0.305), BMI (*p* = 0.988), and parity (*p* = 0.202). In the PE group, gestational age at birth and birth weight were observed to be lower compared to the control group (*p* < 0.001 and *p* < 0.001, respectively). In the PE group, 42.5% of newborns required admission to the NICU, and approximately 41.4% stayed in the NICU for more than 2 days. In the control group, these rates were 8.6% and 8.1%, respectively (*p* < 0.001 and *p* < 0.001, respectively). Additionally, an Apgar score of ≤ 7 at 5 min, the development of RDS, the need for inotropes, sepsis, the requirement for mechanical ventilation, and neonatal acidosis were more frequent in the PE group compared to the control group (all *p* < 0.001). The frequency of CAPO was 44.1% in the PE group and 8.1% in the control group (*p* < 0.001).

**Table 1:** Comparison of maternal demographic characteristics and neonatal outcomes between preeclamptic and control groups

Variables	PE (*n* = 186)	Control (*n* = 186)	*p*-value
Maternal Demographic and Clinical Parameters			
Maternal Age (years)	29 (24–34)	28 (25–31)	0.305^a^
BMI (kg/m^2^)	30.00 (27.00–34.00)	32.00 (26.00–34.00)	0.988^a^
Gravidity	2 (1–3)	2 (1–3)	0.202^a^
Parity	1 (0–2)	1 (0–2)	0.097^a^
Gestational age at sampling (weeks)	36.0 (32.6–37.6)	36.0 (35.0–37.0)	0.176^a^
Placental Abruption	4 (2.2%)	-	0.123^b^
Requirement for Blood Product Transfusion	14 (7.5%)	7 (3.8%)	0.116^b^
ICU Admission	5 (2.7%)	-	0.061^b^
Perinatal Outcomes			
Gestational Age at Birth (weeks)	36 (33–38)	38 (37–39)	< 0.001^a^
Birth Weight (g)	2425 (1650–3030)	3250 (3020–3600)	< 0.001^a^
Apgar Score at 1st Minute	8 (7–9)	9 (9–9)	< 0.001^a^
Apgar Score at 5th Minute	9 (8–10)	10 (10–10)	< 0.001^a^
NICU Admission	79 (42.5%)	16 (8.6%)	< 0.001^b^
Duration of NICU Admission (days)	14 (7–25)	5 (4–5)	< 0.001^a^
NICU Admission > 2 days	77 (41.4%)	15 (8.1%)	< 0.001^b^
5th Minute Apgar Score ≤ 7	28 (15.1%)	-	< 0.001^b^
RDS	55 (29.6%)	2 (1.1%)	< 0.001^b^
Inotrope Requirement	17 (9.1%)	-	< 0.001^b^
Sepsis	17 (9.1%)	-	< 0.001^b^
Need for Mechanical Ventilation	19 (10.2%)	-	< 0.001^b^
Neonatal Asidosis	47 (25.3%)	3 (1.6%)	< 0.001^b^
CAPO	82 (44.1%)	15 (8.1%)	< 0.001^b^

*Abbreviations: PE* Preeclampsia, *BMI* Body Mass Index, *ICU* Intensive Care Unit, *NICU* Neonatal Intensive Care Unit, *RDS* Respiratory Distress Syndrome, *CAPO* Composite Adverse Perinatal Outcome

^a^The Mann–Whitney U test was used for comparisons between groups. Data are presented as median (25–75 percentile)

^b^Categorical variables were compared using the chi-square or Fisher’s exact test, as appropriate. Results are shown as n (%)

When comparing laboratory and inflammatory parameters between the groups (Table 2), neutrophil counts and BUN levels were higher in the PE group (*p* < 0.001 and *p* < 0.001, respectively). In contrast, albumin levels were lower (*p* < 0.001). However, no significant differences were found between the groups in terms of fibrinogen and creatinine levels (*p* = 0.136 and *p* = 0.399, respectively). When the groups were examined in terms of inflammatory indices, the levels of NAR, sACR, FAR, and BAR were significantly higher in the PE group compared to the control group (*p* < 0.001 for all). However, no significant difference was observed in terms of HALP scores (*p* = 0.495).

**Table 2:** Comparison of laboratory parameters and inflammatory indices between preeclamptic and control groups

Variables	PE (*n* = 186)	Control (*n* = 186)	*p*-value*
Hemoglobin (g/dL)	12.0 (11.1–12.9)	11.7 (10.8–12.3)	0.010
WBC count (10³/µL)	12,205 (9840–15340)	10,655 (9600–12240)	< 0.001
Platelet count (10³/µL)	222 (172–278)	254 (216–286)	< 0.001
Neutrophil count (10³/µL)	10.10 (7.60-13.61)	7.76 (6.95–9.11)	< 0.001
Lymphocyte count (10³/µL)	1.80 (1.27–2.34)	2.08 (1.59–2.33)	0.011
Albumin (g/L)	33.0 (29.0–36.0)	37.8 (36.2–38.3)	< 0.001
Fibrinogen (mg/dL)	476 (408–551)	483 (434–538)	0.136
AST U/L	21 (16–34)	17 (15–20)	< 0.001
ALT U/L	14 (10–22)	10 (9–15)	< 0.001
BUN (mg/dL)	19.50 (14.00–25.00)	15.25 (11.50–16.70)	< 0.001
Creatinin (mg/dL)	0.50 (0.40–0.60)	0.51 (0.46–0.56)	0.399
NAR	0.31 (0.22–0.45)	0.22 (0.18–0.24)	< 0.001
sACR	6.00 (4.86–7.75)	7.28 (6.49–7.96)	< 0.001
FAR	14.24 (12.16–17.46)	12.92 (11.58–14.50)	< 0.001
BAR	0.60 (0.40–0.83)	0.40 (0.31–0.45)	< 0.001
HALP	32.99 (20.74–49.32)	34.18 (25.66–45.25)	0.495

*Abbreviations: PE* Preeclampsia, *WBC* White Blood Cell, *AST* Aspartate Aminotransferase, *ALT* Alanine Aminotransferase, *BUN* Blood Urea Nitrogen, *PT* Prothrombin Time, *aPTT* Activated Partial Thromboplastin Time, *INR* International Normalized Ratio, *NLR* Neutrophil-to-Lymphocyte Ratio, *PLR* Platelet-to-Lymphocyte Ratio, *NAR* Neutrophil-to-Albumin Ratio, *sACR* Serum Albumin-to-Creatinine Ratio, *FAR* Fibrinogen-to-Albumin Ratio, *BAR* Blood Urea Nitrogen-to-Albumin Ratio, *HALP* Hemoglobin, Albumin, Lymphocyte, Platelet score, *SII* Systemic Immune-Inflammation Index

*The Mann–Whitney U test was used for comparisons between groups. Data are presented as median (25–75 percentile)

In the PE group, CAPO developed in 82 of patients, while it was absent in 104. When comparing the demographic, clinical, laboratory, and inflammatory parameters of PE patients with and without CAPO (Table 3), a higher incidence of severe preeclampsia was observed in the CAPO group (86.6% vs. 61.5%, *p* < 0.001). When laboratory parameters were examined, the neutrophil count, BUN, and creatinine levels were higher in the CAPO group compared to the non-CAPO group (*p* = 0.043, *p* < 0.001, and *p* = 0.004, respectively). In the CAPO group, NAR, BAR, and HALP scores were higher (*p* = 0.005, *p* < 0.001, *p* = 0.018, respectively), while sACR levels were lower (*p* < 0.001). FAR levels were similar between the two groups (*p* = 0.566).

**Table 3:** Comparison of maternal characteristics and laboratory parameters between preeclamptic women with and without CAPO

Variables	CAPO (*n* = 82)	Non-CAPO (*n* = 104)	*p*-value*
Maternal Age (years)	28 (23–33)	29 (24–34)	0.126^a^
BMI (kg/m^2^)	29.00 (25.00–32.00)	31.00 (28.00–34.00)	0.017^a^
Severe Preeclampsia	71 (86.6%)	64 (61.5%)	< 0.001^b^
Gestational Age at Delivery and Blood Sampling (weeks)	32.6 (30.0-35.1)	37.2 (36.2–38.4)	< 0.001^a^
Birth Weight (g)	1585 (1120–2150)	2900 (2395–3265)	< 0.001^a^
Laboratory Parameters			
Hemoglobin (g/dL)	12.2 (11.4–13.0)	11.6 (11.0-12.8)	0.036^a^
WBC count (10³/µL)	12,990 (10130–16500)	11,910 (9515–14875)	0.046^a^
Platelet count (10³/µL)	212 (133–260)	227 (184–305)	0.005^a^
Neutrophil count (10³/µL)	10.63 (8.05–14.88)	9.38 (7.29–12.34)	0.043^a^
Lymphocyte count (10³/µL)	1.80 (1.40–2.43)	1.80 (1.21–2.26)	0.447^a^
Albumin (g/L)	31.0 (28.0–36.0)	33.5 (30.5–37.0)	0.003^a^
Fibrinogen (mg/dL)	468 (289–542)	479 (424–554)	0.242^a^
AST U/L	22 (16–48)	20 (15–28)	0.017^a^
ALT U/L	15 (11–38)	12 (9–19)	0.005^a^
BUN (mg/dL)	23.00 (18.00–29.00)	17.00 (13.00–21.00)	< 0.001^a^
Creatinin (mg/dL)	0.60 (0.50–0.70)	0.50 (0.40–0.60)	0.004^a^
NAR	0.38 (0.24–0.49)	0.30 (0.22–0.37)	0.005^a^
sACR	5.50 (4.57–6.80)	6.40 (5.31–8.25)	< 0.001^a^
FAR	14.50 (11.51–18.57)	14.03 (12.47–16.89)	0.566^a^
BAR	0.76 (0.52-1.00)	0.51 (0.37–0.69)	< 0.001^a^
HALP	37.16 (23.41–52.90)	29.58 (19.51–44.90)	0.018^a^

*Abbreviations: PE* Preeclampsia, *BMI* Body Mass Index, *WBC* White Blood Cell, *AST* Aspartate Aminotransferase, *ALT* Alanine Aminotransferase, *BUN* Blood Urea Nitrogen, *PT* Prothrombin Time, *aPTT* Activated Partial Thromboplastin Time, *INR* International Normalized Ratio, *NLR* Neutrophil-to-Lymphocyte Ratio, *PLR* Platelet-to-Lymphocyte Ratio, *NAR* Neutrophil-to-Albumin Ratio, *sACR* Serum Albumin-to-Creatinine, *FAR* Fibrinogen-to-Albumin Ratio, *BAR* Blood Urea Nitrogen-to-Albumin Ratio, *HALP* Hemoglobin, Albumin, Lymphocyte, Platelet score, *SII* Systemic Immune-Inflammation Index

^a^The Mann–Whitney U test was used for comparisons between groups. Data are presented as median (25–75 percentile)

^b^Categorical variables were compared using the chi-square or Fisher’s exact test, as appropriate. Results are shown as n (%)

In the logistic regression analysis evaluating indices associated with CAPO in the PE cohort, BMI (OR 0.943; *p* = 0.026), Gestational Age at Birth / Blood Sampling (weeks) (OR 0.637; *p* < 0.001), and severe preeclampsia (OR 4.034; *p* < 0.001) were associated with CAPO in the univariable model. NAR (OR 1.186, *p* = 0.043), sACR (OR 0.801, *p* = 0.001), BAR (OR 1.316, *p* < 0.001), and HALP (OR 1.012, *p* = 0.020) were found to be associated with CAPO. In the multivariable model, when BMI, Gestational Age at Birth/ blood Sampling (weeks), and preeclampsia severity were controlled for, only BAR was found to be independently associated with the development of CAPO (aOR 0.874, *p* = 0.015), while the other albumin-related indices lost their significance. These results are shown in Table 4.

**Table 4:** Logistic regression analysis of maternal and inflammatory parameters associated with CAPO in the preeclampsia cohort

Variables	Univariable			Multivariable		
	OR	95% CI	*p*-value	aOR	95% CI	*p*-value
BMI (kg/m^2^)	0.943	0.896–0.993	0.026			
Gestational Age at Birth / Blood Sampling (weeks)	0.637	0.555–0.730	< 0.001			
Severity of Preeclampsia	4.034	1.910–8.522	< 0.001			
NAR*	1.186	1.006–1.398	0.043	1.058	0.889–1.259	0.528
sACR	0.801	0.699–0.916	0.001	0.874	0.745–1.025	0.097
BAR*	1.316	1.169–1.481	< 0.001	1.157	1.029–1.301	0.015
HALP	1.012	1.002–1.022	0.020	1.009	0.997–1.022	0.146

Due to NAR, sACR, BAR, and HALP share a common component (albumin), each was analyzed separately in the multivariable logistic regression models, adjusted for BMI (kg/m²), Gestational Age at Birth / Blood Sampling (weeks), and severity of preeclampsia

*Abbreviations: OR* Odds Ratio, *CI* Confidence Interval, *aOR* Adjusted Odds Ratio, *BMI* Body Mass Index, *CAPO* Composite Adverse Perinatal Outcome, *NAR* Neutrophil-to-Albumin Ratio, *sACR* Serum Albumin-to-Creatinine Ratio, *BAR* Blood Urea Nitrogen-to-Albumin Ratio, *HALP* Hemoglobin, Albumin, Lymphocyte, Platelet score

*In both models, NAR and BAR values were multiplied by 10 before logistic regression to address excessively wide odds ratios and confidence intervals, thereby enhancing the interpretability of the estimates without influencing their statistical significance

**Fig. 2:**
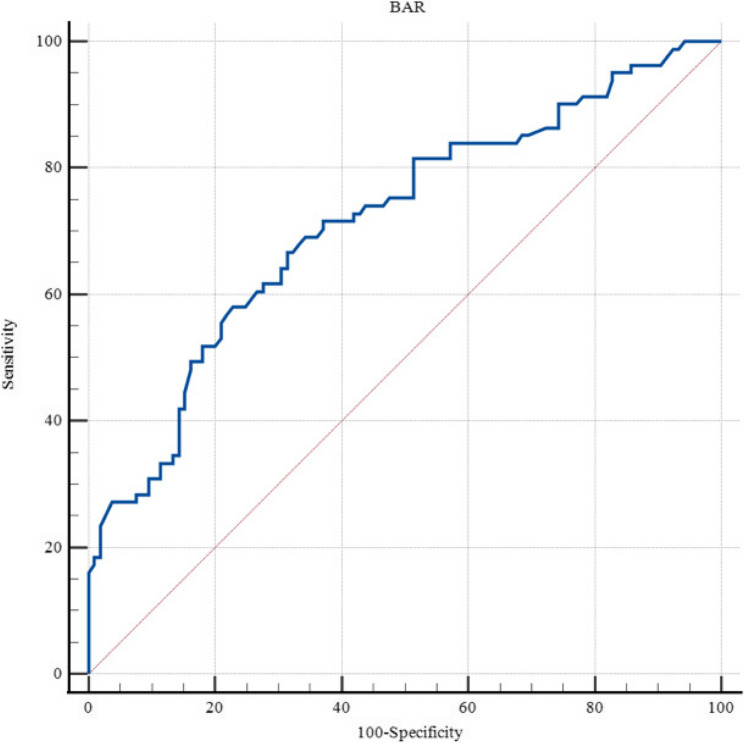
ROC curve analysis of the predictive performance of the BAR for CAPO in the preeclampsia cohort

Several parameters demonstrated significant differences in univariable analyses; however, only the BAR remained a significant independent predictor of CAPO in the preeclampsia cohort according to the multivariable logistic regression model. Therefore, the ROC analysis was conducted exclusively for BAR to assess its discriminative ability as shown in Table 5; Fig. 2. BAR (cut-off value > 0.61, AUC = 0.718, sensitivity = 67.1%, specificity = 69.2%, *p* < 0.001) demonstrated a significant ability to predict CAPO, achieving an AUC value of 0.718.

**Table 5:** ROC analysis of BAR for predicting CAPO in preeclamptic patients

Variables	Cut-off	AUC	95% CI	Sensitivity (%)	Specificity (%)	LR^+^	LR^−^	*p*-value
BAR	> 0.61	0.718	0.648–0.781	67.1	69.2	2.18	0.48	< 0.001

*Abbreviations: ROC* Receiver Operating Characteristic, *AUC* Area Under the Curve, *CI* Confidence Interval, *LR* Likelihood Ratio, *BAR* Blood Urea Nitrogen-to-Albumin Ratio

## Discussion

This research evaluated albumin-derived inflammatory markers (NAR, FAR, sACR, HALP, and BAR scores) in pregnancies complicated by preeclampsia. Our results indicated that NAR, FAR, and BAR levels were significantly increased in the preeclamptic cohort, whereas sACR levels were reduced, and HALP scores showed no significant differences between groups. In preeclamptic cases exhibiting CAPO, levels of NAR, BAR, and HALP were elevated, while sACR levels were reduced. In multivariable logistic regression analysis, BAR remained the sole independent predictor for CAPO (aOR 1.157, *p* = 0.015).

Inflammation and immune dysregulation play central roles in the pathogenesis of preeclampsia [12, 16, 23]. Endothelial injury and inflammatory activation promote the release of proinflammatory cytokines, leading to neutrophil activation, oxidative stress, and coagulation imbalance [12, 24, 25]. These mechanisms may impair placental perfusion, contribute to fetal hypoxia, and increase the risk of adverse neonatal outcomes [8–10, 12]. Therefore, inexpensive and readily available inflammatory biomarkers may be useful for risk assessment in preeclamptic pregnancies.

BAR serves as a marker that concurrently represents two essential pathophysiological processes. BUN is an indirect marker of the accumulation of nitrogenous metabolites and renal perfusion; albumin, on the other hand, is a negative acute phase reactant that is affected by changes in inflammation and nutritional status. In the literature, BAR has generally been studied in critical illness conditions. For example, Dündar et al. reported that high BAR levels in elderly patients presenting to the emergency department were independently associated with in-hospital mortality [26]. Huang et al. [27] have shown that high BAR values in cases of COVID-19 infection are associated with increased mortality [25]. These studies emphasize that BAR may be an easily accessible prognostic biomarker reflecting both renal function and systemic inflammatory burden. The existing literature on BAR in obstetric practice is limited. Only Bayrak et al. have examined BAR levels in instances of placental abruption, indicating markedly increased levels compared to healthy pregnancies [15]. To our knowledge, this is the first study to evaluate BAR in preeclampsia. We found that BAR levels were significantly higher in preeclamptic pregnancies than in healthy controls and were also independently associated with CAPO. ROC analysis further supported its discriminatory performance (AUC = 0.718, cut-off > 0.61, sensitivity 67.1%, specificity 69.2%). Elevated BAR levels in preeclampsia may reflect the combined effects of endothelial dysfunction, intravascular volume depletion, and impaired renal perfusion. Overall, these findings suggest that BAR may be a clinically useful marker for identifying preeclamptic patients at increased risk of adverse neonatal outcomes.

FAR is an easily measurable marker that reflects both inflammation and coagulation. Previous studies have shown that elevated FAR levels are associated with systemic inflammatory activity [28, 29]. In obstetric populations, Kından et al. reported significantly higher FAR levels in HELLP syndrome than in healthy controls, with high diagnostic accuracy [30]. Similarly, Ren et al. showed that FAR levels were elevated in preeclamptic pregnancies and positively correlated with disease severity [31]. In our study, FAR was also significantly higher in the preeclampsia group, although it was not independently associated with CAPO.

NAR combines two features of the inflammatory response: an increased neutrophil count and a decreased albumin level, a negative acute-phase reactant. It has therefore been proposed as a marker of systemic inflammation. Previous studies have evaluated NAR in several obstetric conditions [20, 30, 32]. Kından et al. reported that NAR had diagnostic value for HELLP syndrome and adverse maternal and neonatal outcomes [30]. Biçer et al. also showed that NAR was elevated in preeclamptic pregnancies from the first trimester, particularly in early-onset cases [20]. Consistent with these findings, NAR was significantly higher both in preeclampsia and in PE cases complicated by CAPO in our cohort. However, after adjustment for maternal BMI, gestational age at delivery, and disease severity, NAR was no longer independently associated with CAPO. This suggests that systemic inflammation may contribute to disease activity, but that NAR alone may be insufficient to predict adverse perinatal outcomes when renal dysfunction and placental perfusion are also involved.

sACR serves as a composite measure of inflammatory response and renal function. A survey of the literature indicates that it has been examined more extensively in cardiac disorders [33–35]. Artaç et al. indicated that low sACR correlated with mortality in myocardial infarction patients [34], whereas Baş et al. established that diminished sACR was linked to chemotherapy-induced cardiotoxicity [35]. Data on sACR in obstetric practice are limited. Bayrak et al. reported that sACR levels were significantly lower in placental abruption than in healthy pregnancies [15]. In our study, sACR was also significantly lower in preeclamptic pregnancies and in PE cases with CAPO. However, this association did not remain significant after multivariable adjustment. Although both BAR and sACR combine albumin with a renal function parameter, only BAR remained independently associated with CAPO. This difference may be related to the distinct physiological behavior of BUN and creatinine. BUN generally rises earlier and more prominently than creatinine, especially in prerenal states and systemic hypoperfusion, whereas creatinine often increases only after more advanced renal impairment has developed [36]. Therefore, sACR may be less sensitive than BAR in capturing early renal-endothelial dysfunction in preeclampsia.

HALP is a composite index reflecting inflammation, hematologic response, and nutritional status [37]. In obstetric studies, Polat et al. reported that first-trimester HALP was not significantly associated with the prediction of preeclampsia [38]. Seyhanlı et al. found lower HALP scores in late-onset fetal growth restriction [39] reported that first trimester HALP scores were significantly lower in cases of late-onset fetal growth restriction. Ağaoğlu et al. [22] reported that HALP scores were lower in patients who delivered preterm and could be a clinically useful parameter. Kından et al. [30] reported that HALP scores were lower in patients who delivered preterm and could be a clinically useful parameter. In our study, HALP scores did not show a significant difference between preeclamptic and healthy pregnant women; however, they were significantly higher in cases of CAPO. Despite this, they did not remain an independent predictor in multivariable analysis.

Our study has several limitations. First, its retrospective single-center design limits the generalizability of the findings. Second, serum parameters were evaluated at only one time point, namely at delivery in the preeclampsia group; therefore, dynamic changes in these biomarkers throughout the course of the disease could not be assessed. Therefore, these findings should be interpreted as reflecting cross-sectional associations at delivery, which limits the predictive interpretation of the evaluated biomarkers. Another important limitation is the absence of complete and reliably retrievable data on antenatal corticosteroid (ACS) and magnesium sulfate (MgSO₄) exposure in the retrospective medical records. Because a substantial proportion of patients delivered preterm, both treatments may have influenced inflammatory pathways, hematologic indices, and neonatal outcomes [40, 41]. Therefore, their potential confounding effect on the association between albumin-related inflammatory indices and composite adverse perinatal outcomes could not be evaluated in the present study. In addition, other potential confounders such as maternal nutritional status, subclinical infection, baseline renal function, and concomitant antihypertensive treatment were not fully controlled. Another limitation is that CAPO included outcome components with varying clinical severity, which introduced heterogeneity into the composite endpoint and should be considered when interpreting the findings. The study also does not include an external validation cohort or direct comparison with existing risk models. Therefore, prospective multicenter studies with serial biochemical measurements, standardized treatment recording, and external validation are needed to confirm the predictive value of BAR and clarify its place in clinical risk stratification.

## Conclusion

In conclusion, in this study all albumin-based indices evaluated in preeclamptic pregnancies showed significant differences compared to healthy controls. However, only BAR remained an independent predictor in the assessment of adverse perinatal outcomes. This finding suggests that BAR could be considered as a helpful biomarker for identifying high-risk patients in terms of adverse neonatal prognosis in preeclamptic pregnancies. Being an easily measurable, low-cost parameter in routine laboratories increases its potential value in clinical practice. Large-scale, multicenter, and prospective studies are required to validate these findings.

## Data Availability

Due to hospital policies, patient data and study materials cannot be shared. However, the data are available from the corresponding author upon reasonable request.

## Abbreviations

PE: Preeclampsia
CAPO: Composite Adverse Perinatal Outcome
NICU: Neonatal Intensive Care Unit
RDS: Respiratory Distress Syndrome
BUN: Blood Urea Nitrogen
NAR: Neutrophil-to-Albumin Ratio
NPAR: Neutrophil Percentage-to-Albumin Ratio
FAR: Fibrinogen-to-Albumin Ratio
BAR: Blood Urea Nitrogen-to-Albumin Ratio
sACR: Serum Albumin-to-Creatinine Ratio
HALP: Hemoglobin–Albumin–Lymphocyte–Platelet Score
BMI: Body Mass Index
AUC: Area Under the Curve
OR: Odds Ratio
aOR: Adjusted Odds Ratio
ROC: Receiver Operating Characteristic
ACOG: American College of Obstetricians and Gynecologists
ICU: Intensive Care Unit
